# Digitalis in Delirium Tremens

**Published:** 1871-04

**Authors:** 


Digitalis in Delirium Tremens.—Dr. A. Wiltshire records (*Lancet*, Aug. 27th, 1870) five cases of delirium tremens successfully treated by half-ounce doses of tincture of digitalis. He states that Dr. Russell Reynolds, to whom he had communicated these cases, suggested that possibly the good effect of the half-ounce of tincture of digitalis might be owing to the alcohol it contained, and not to the digitalis; and he recommended that if I had an opportunity of doing so, it would be well to try the effect of half an ounce of proof spirit. Dr. Reynolds also suggested that, if I found it necessary to resort to digitalis, it would be desirable to give a watery infusion of the drug, using a dose of it equal in strength to half an ounce of the tincture. It so happened that, shortly after this conversation, Case 3 again came under my care, with another severe attack of delirium tremens. I felt it to be my duty to give him the benefit of ordinary treatment at the outset, as I had done in the first attack for which I had attended him; but the result was equally unsatisfactory, and ultimately, I judged it to be necessary to give him digitalis again. But before doing so, I determined to put into practice Dr. Reynold's suggestion. I accordingly gave half an ounce of proof spirit; and although for a considerable time I

carefully watched the patient after its administration, I could detect no change whatever either in the pulse, respiration, or mental condition. Finding the result of this experiment to be purely negative, I resorted to the half-ounce dose of tincture of digitalis, as I found a difficulty in procuring good digitalis leaves wherewith to make a watery infusion. This, happily, acted as efficiently as a like dose had done on the first occasion; and the phenoma observed after its administration were of precisely the same character—that is to say, the pulse fell in frequency while it gained in volume and strength, the breathing became more tranquill, and the patient fell asleep within twenty minutes of taking the drug. After some hours' sleep he awoke cured.

No bad or dangerous effects were observed in either of the cases narrated, although, I confess, that the marked decrease in the frequency of the pulse at first made me uneasy; and, had I not recognized that, while it fell in frequency, it gained remarkably both in force and in volume, I should have been alarmed.

It will be observed that my patients were young, or comparatively young, persons; for I am averse to treating the aged by means of large doses of digitalis, being, in truth, as it were instinctively, afraid to do so. And, even in the young, I was and still am loth to resort to so powerful, not to say dangerous, a remedy without, in the first instance, having given the patient the benefit of what is regarded as more orthodox treatment, which, undoubtedly, succeds in the majority of cases.—*Ibid.*

